# The Possible Role of NLRP3 Inflammasome in Depression and Myocardial Infarction Comorbidity

**DOI:** 10.3390/jpm13091295

**Published:** 2023-08-25

**Authors:** Erensu Baysak, Cagan Yildirim, Nurten Sayar, Mustafa Kemal Sayar, Angelos Halaris, Feyza Aricioglu

**Affiliations:** 1 Department of Psychiatry, School of Medicine, Marmara University, Istanbul 34854, Turkey; 2 Department of Cardiology, School of Medicine, Marmara University, Istanbul 34854, Turkey; 3 Department of Psychiatry and Behavioral Neurosciences, Stritch School of Medicine, Loyola University Medical Center, Loyola University Chicago, Maywood, IL 60153, USA; 4 Department of Pharmacology, School of Dentistry and Institute of Health Sciences, Marmara University, Istanbul 34865, Turkey

**Keywords:** depression, *hs*CRP, myocardial infarction, NLRP3, NOD-like receptor protein 3

## Abstract

It is well-established that cardiovascular disease and depression are highly comorbid. This study aimed to assess the possible role of the NOD-like receptor protein 3 (NLRP3) inflammasome pathway and the high-sensitivity C-reactive protein (*hs*CRP) in patients with incident myocardial infarction in the presence or absence of depression. Sixty-eight consecutive patients with incident ST-elevation myocardial infarction and twenty healthy subjects were included. The patients were assessed using the Structured Clinical Interview for DSM-5 Disorders—Clinician Version during their 1–4-day-long hospitalization and were divided into two groups: with and without comorbid depression. Blood samples for the determination of NLRP3, interleukin-18 (IL-18), interleukin-1β (IL-1β), and *hs*CRP levels were analyzed using ELISA. NLRP3, IL-1β, IL-18, and *hs*CRP levels were significantly higher in myocardial infarction patients compared to the healthy group (*p* = 0.02, *p* < 0.001, *p* < 0.001, and *p* < 0.001, respectively). No significant difference was found between the myocardial groups with and without depression. However, in the logistic regression analysis, the NLRP3 variable in myocardial infarction patients was found to have a significant contribution to the likelihood of depression (*p* = 0.015, OR = 1.72, and CI = 1.11–2.66). The likelihood of depression is associated with increasing NLRP3 levels in myocardial infarction patients. However, this potential role should be further explored in a larger sample.

## 1. Introduction

Cardiovascular disease (CVD) and depression are among the leading health problems worldwide [1]. It is well-known that CVD and depression are highly comorbid, and studies suggest that this relationship is bidirectional [2]. Approximately 35–40% of patients with CVD report depressive symptoms, and depressed individuals have a 1.5 times higher risk of developing CVD than non-depressed individuals [3]. Many large-scale prospective studies showed that depression and even subthreshold depressive symptoms are independent risk factors for CVD [4,5,6,7]. The link between depression and CVD was previously referred to as “heartache and heartbreak” [8]. Depression is associated with an increased risk of all-cause and CVD mortality [9]. Thus, it is important to successfully screen for and treat depression to decrease morbidity and mortality in patients with CVD [10]. The accumulating evidence from recent studies has demonstrated that the immune system plays a role in the pathophysiology of depression [11,12]. Similarly, mechanisms associated with inflammation in the etiology of CVD have attracted attention, and we now know that, besides classical risk factors, inflammation is associated with cardiovascular risk, and atherosclerosis is not a simple process that starts with lipid deposition, as previously thought, but is mediated by inflammation [13]. 

High-sensitivity C-reactive protein (*hs*CRP) is a well-known biomarker for assessing cardiovascular risk [14]. At the same time, *hs*CRP blood levels are reportedly higher, at least in some major depressive disorder (MDD) patients compared to healthy individuals, and are associated with the inflammation hypothesis of depression [15]. 

Recent studies on the relationship between depression and systemic comorbid diseases focused on the possible role of the nucleotide-binding oligomerization domain (NOD)-like receptor protein 3 (NLRP3) inflammasome underlying their pathophysiology [16,17]. In 2002, the term “inflammasome” was first used by Martinon [18] for a multiprotein complex that leads to the secretion of bioactive interleukin-1β and interleukin-18 as well as the induction of inflammatory cell death, termed pyroptosis [19]. This complex comprises NLRP3 itself as an intracellular sensor, the adaptor protein apoptosis-associated speck-like protein containing a caspase-recruitment domain (ASC), and the effector enzyme, pro-caspase-1 [20]. The NLRP3 inflammasome is activated by a range of danger and stress signals including ion fluxes, lysosomal leakage, mitochondrial dysfunction, and reactive oxygen species production [21]. A two-step process is required for its activation [19]. A “priming” signal induces the transcription of the components of the inflammasome (NLRP3, pro-IL-1β, and pro-IL-18). A “triggering” signal is necessary for the assembly of the inflammasome [22]. 

The NLRP3 inflammasome is highly expressed in innate immune cells, including macrophages and neutrophils, and is also expressed in nonimmune cells, including endothelial cells, cardiomyocytes, fibroblasts, and epithelial cells [23]. The accumulating evidence has indicated that the NLRP3 inflammasome plays a key role in the pathogenesis of chronic inflammatory diseases and metabolic disorders, including obesity, hypertension, diabetes, atherosclerosis, depression, stroke, and cancer [21,24]. 

Oxidative stress, mitochondrial dysfunction, and immune activation may also trigger a common signaling pathway of NLRP3 inflammasome activation, thereby exacerbating endothelial dysfunction [21]. NLRP3-dependent pyroptosis and endothelial dysfunction might be common mechanisms for CVD and depression comorbidity [21,25,26,27]. Certain drugs (i.e., statins, hypoglycemic agents, and other anti-inflammatory drugs) can improve vascular dysfunction by inhibiting the NLRP3 inflammasome [21]. It is noteworthy that this same class of drugs demonstrated antidepressant-like effects [28,29,30]. Some antidepressants such as fluoxetine, paroxetine, mirtazapine exerted potential anti-pyroptosis activity by inhibiting the expression of NLRP3 [31]. 

NLRP3 inflammasome plays a pivotal role in the progression of atherosclerosis, coronary artery disease (CAD), and myocardial ischemia–reperfusion (I/R) injury [32]. Toldo et al. conducted a comprehensive review of the NLRP3 inflammasome in acute myocardial infarction [33]. NLRP3, an extensively studied inflammasome sensor in the heart, is activated by cell debris during AMI. Within the context of AMI, toll-like receptors sense DAMPs and prime the cells to enhance the transcription and translation of inflammasome components. The increase in extracellular ATP, lysosomal destabilization, ineffective mitophagy, and autophagy contribute to NLRP3 activation in AMI. This inflammatory response subsequently exacerbates myocardial damage. The inhibition of NLRP3 inflammasome activation after myocardial infarction proves beneficial in reducing infarct size and preserving cardiac function [33]. A recent study reported that plasma NLRP3 protein levels were 1.77 (0.7–5.79) ng/mL in patients with myocardial infarction and 0.77 (0.4–0.9) ng/mL in the control group [34]. The authors stated that the NLRP3 plasma protein level could be a potential biomarker for MI with the cut-off point at 0.75 ng/mL. Wang et al. measured NLRP3, IL-1β, and IL-18 levels in patients with acute coronary syndrome (ACS), and there was a correlation between the severity of ACS, as assessed by the Gensini score, and the levels of these parameters [35]. NLRP3 mRNA levels decreased with time after acute MI; however, they were higher than in healthy controls at every time point of the measurements. 

It was proposed that the NLRP3 inflammasome translates psychologically stressful stimuli into inflammatory responses and depressive-like behavior [30]. However, clinical data regarding the involvement of NLRP3 in patients with depression are limited. A few studies reported that NLRP3 protein levels are increased in the peripheral blood mononuclear cells of patients with depression compared to non-depressed subjects [31,36,37]. The potential mechanisms underlying the activation of the NLRP3 inflammasome in depression are the recognition of the pathogen-associated molecular patterns (PAMPs) of danger-associated molecular patterns (DAMPs) by toll-like receptors, K^+^ outward flow via the purinergic receptor P2X7R, increased intracellular Ca^2+^ levels, endoplasmic reticulum stress, increased reactive oxygen species generation, and autophagy dysfunction [38].

The possible *bridge* role that NLRP3 may play in the comorbidity of these two disease entities has not been investigated thus far. In this study, we aimed to investigate the possible role of the NLRP3 inflammasome and *hs*CRP in this comorbidity and to evaluate the relationship of these biomarkers with the presence and severity of comorbid depression in patients with acute myocardial infarction.

## 2. Material and Methods

### 2.1. Participants

Sixty-eight patients between the ages of 18 and 55 who were referred to the Marmara University Pendik Research and Training Hospital Cardiology Department with incidental ST-segment elevation myocardial infarction (STEMI) were included in the study. Patients were diagnosed with STEMI if they applied to the emergency department with anginal symptoms, ST-segment elevation in electrocardiogram (>2.5 mm ST elevation at the J point in leads V2-3 for male patients younger than 40 years old, >2 mm for male patients older than 40 years old, and >1.5 mm for female patients regardless of age as well as >1 mm ST elevation in any other two contiguous leads regardless of age and gender [39]). The patients underwent primary percutaneous coronary intervention (PCI) and were transferred to the coronary intensive care unit. The sample size estimation led to a minimum requirement of 20 participants per group (Cohen’s d = 0.915).

After the immediate cardiological interventions, both clinical interviews and psychiatric evaluations were performed within 1-4 days of patients’ hospitalization following their hospital admission for STEMI. During the clinical evaluation, patients were diagnosed with Major Depressive Disorder (MDD) using the validated Turkish version of Structured Clinical Interview for DSM-5 Disorders—Clinician Version (SCID-5-CV) [40] and were divided into two groups: myocardial infarction with depression (MID) or without depression (MI). Patients were also evaluated using the validated Turkish version of Beck’s Depression Inventory (BDI) [41], the validated Turkish version of the 17-item Hamilton Depression Rating Scale (HDRS) [42], the Sheehan Disability Scale (SDS) [43], and the validated Turkish version of the Perceived Stress Scale (PSS) [44]. BDI is a 21-item, self-reported rating inventory to assess the intensity of depressive symptoms, with a total score ranging from 0 to 63. Higher scores reflect more depressive symptoms, and the calculated cut-off score for identifying significant depression is 7 [41]. The HDRS is a clinician-administered depression assessment scale. Scores of 0–7 are accepted to be within the normal range, 8–16 suggest mild depression, 17–23 suggest moderate depression, and scores above 24 are indicative of severe depression [45]. The SDS is a 3-item scale that assesses functional impairments associated with work/school, social life and leisure activities, and family life and home responsibilities, with higher scores indicating greater functional impairment. PSS is a 14-item self-report measure intended to evaluate participants’ perception of stress. The total score ranges from 0 to 56, with higher scores indicating increased levels of perceived stress. The Gensini score was calculated by cardiologists to determine the severity of atherosclerosis [46].

All participants with other psychiatric disorders and a prior history of atherosclerotic vascular disease (ASVD) were excluded. The control group was screened for depressed mood and anhedonia (for 1 main criterion for depression diagnoses) according to SCID-5. Additional exclusion criteria were the presence of an autoimmune disorder; severe metabolic syndrome; concurrent treatment with antidepressant, anti-inflammatory, antiviral, antibiotic, or immune-modulating drugs; pregnancy; lactation; and inability to provide informed consent. Information about history of ASVD and depression was obtained from the interview with the patients and the medical records. 

### 2.2. Blood Collection and Serum Isolation

Blood samples from 20 age- and sex-matched healthy subjects (5 females and 15 males) were included in the study. The blood collection was limited to the first 4 days of hospitalization to standardize the factors related to an inflammatory response caused by myocardial infarction itself as well as the anti-inflammatory effects of additional medication (i.e., statins that were prescribed during the hospitalization). During the 1–4-day-long hospitalization of the patients and after an overnight fast, the samples were collected between 08:00 and 10:00 in the morning into a serum separator tube; approximately 10–15 min after clotting, they were centrifuged at 1000× *g* for 15 min, and serum was aliquoted and stored at −80 °C until analyzed. 

### 2.3. Measuring NLRP3, IL-1β, IL-18, and hsCRP Levels

Serum levels of NLRP3 sensor, IL-1β, IL-18, and *hs*CRP were assayed by ELISA kits (Aviva Systems Biology, San Diego, CA, USA; Diaclone SAS, Besançon, France; Thermo Fisher Scientific, Waltham, MA, USA; DRG International Inc., Springfield, NJ, USA, respectively). The assays were performed in accordance with the instructions of the manufacturer. 

### 2.4. Statistical Analyses

Statistical analyses were performed with IBM SPSS 23.0 package program (IBM Corp., Armonk, NY, USA). For statistical significance *p* < 0.05 values were considered. 

Pearson’s chi-square test and Fisher’s exact test were used to analyze the relationships between categorical variables, and Bonferroni correction was made for pairwise comparisons. The assumption of normal distribution was verified with the Shapiro–Wilk test. In the analysis of the difference between the values of the MI and MID groups, the Mann–Whitney U test was used, when it did not fit the normal distribution, and the Student’s t-test was used when it did. The Kruskal–Wallis test was used in the non-parametric comparison of the measurements of the control, MI, and MID groups, and the Bonferroni–Dunn test was used as a post hoc test for significant cases. ANOVA test was used when the assumption of normal distribution was met, and Tukey’s HSD test was used when homogeneity of variance was provided for pairwise comparisons; otherwise, Dunnett’s T3 test was used. The relationship between inflammatory parameters and depression was assessed with binary logistic regression analysis with depression as the dependent variable. Both unadjusted analysis (Model 1) and adjusted analysis (Model 2; adjusted for age, body mass index (BMI), diabetes comorbidity, use of additional medication, Gensini score, duration between myocardial infarction, and blood collection covariates) were conducted.

## 3. Results

This study presents the results from 68 patients with acute myocardial infarction (AMI), MI with depression (MID group), and MI without depression (MI group) and 20 age- and sex-matched healthy subjects (control group). The characteristics of the groups are shown in Table 1. There were no statistical differences among groups according to age, sex, and BMI. Within the MID group, 33.3% (*n* = 8) of the patients had a previous history of depression. Whereas 18.2% (*n* = 8) of the MI group had comorbid diabetes, 37.5% (*n* = 9) of the MID group had comorbid diabetes, but the difference between the two groups was not statistically significant. The detailed medical history of the diabetic patients can be found in Supplementary Table S1. The smoking status in the healthy control group was significantly lower than in the MI and MID groups (*p* = 0.002). The comparison between the MI and MID groups according to depression, disability, and perceived stress scores is shown in Table 1. In the MID group, the mean score of the HDRS was 13.75 ± 2.98, and the mean score of BDI was 18.08 ± 4.57. The scores of all scales were significantly higher in the MID group.

### 3.1. Inflammatory Parameters in MI Patients and Control Group

There was no significant difference between the MI and MID groups in terms of CK-MB, troponin, N-terminal pro-B-type natriuretic peptide (NT-proBNP) levels, Gensini scores, and the number of days between AMI and blood collection (Table 2). 

**Figure 1:**
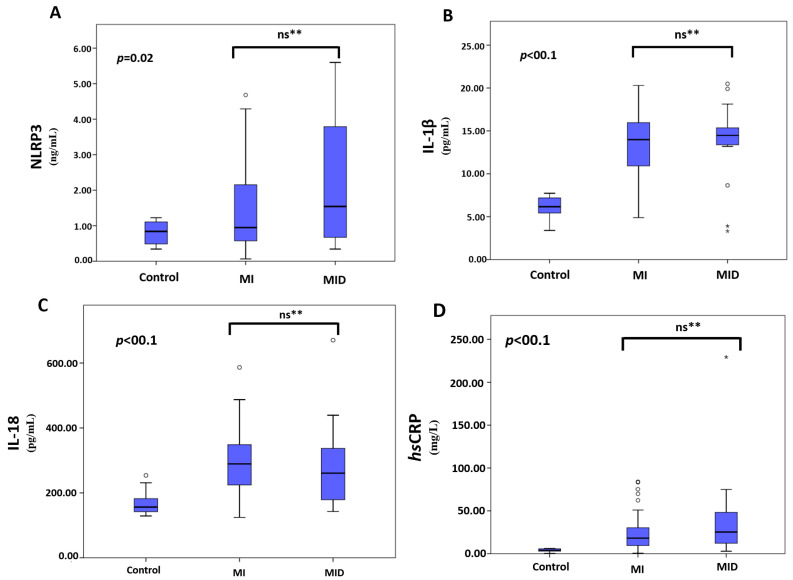
Comparison of NLRP3, IL-1β, IL-18, and *hs*CRP levels of the groups. Panel (**A**–**D**). NLRP3, IL-1β, IL-18, and *hs*CRP serum levels were assessed using ELISA; *n* = 20 for control, *n* = 44 for MI, and *n* = 24 for MID groups. The control group had significantly lower levels for all parameters. However, there were no significant differences for NLRP3, IL-1β, IL-18, and *hs*CRP between the MI and MID groups. ** ns: not significant; ° and *: outliers; MI: myocardial infarction; MID: myocardial infarction with depression.

The comparison of inflammatory parameters among groups is summarized in Table 3 and in Figure 1. There was no significant difference between the MI and MID groups for NLRP3 levels; NLRP3 levels were significantly lower in the healthy control group compared to the other two groups (*p* = 0.02). There was no statistically significant difference between the MI and MID groups for IL-1β, IL-18, and *hs*CRP levels, whereas IL-1β, IL-18, and *hs*CRP levels were significantly *lower* in the healthy control group (*p* < 0.001). Although a significant difference regarding the inflammatory parameters between the MI and MID groups was not detected, there is a noticeable increasing trend from the control group to MID. There was no statistically significant correlation between Gensini score, depression scores, and inflammatory markers in the MI, MID, and healthy control groups, whether diabetes patients were included or excluded (Supplementary Tables S2 and S3). 

### 3.2. Comparison of Inflammatory Parameters in the Presence of Type 2 Diabetes

There were *n* = 8 (18.2%) comorbid diabetes diagnoses in the MI group and *n* = 9 (37.5%) in the MID group (Supplementary Table S4). There were lower levels of all inflammatory parameters in the presence of diabetes, and this was found to be statistically significant for IL-18 and *hs*CRP in the MID group (*p* = 0.035 and *p* = 0.021, respectively). 

### 3.3. Increased NLRP3 Levels Are Associated with the Likelihood of Depression in Acute Myocardial Infarction Patients

To evaluate the relationship between NLRP3, IL-1β, IL-18, and *hs*CRP levels and depression in patients with myocardial infarction, binary logistic analysis with depression as the dependent variable was performed. The potential confounders considered in inflammation studies include age, BMI, additional inflammatory diseases, other medications that possibly interfere with inflammatory pathways, and the severity of inflammation. In a clinical setting, it was not possible to eliminate all of these factors. However, we tried to decrease the heterogeneity in a small sample of study participants by limiting the patient group to STEMI patients, calculating the severity of atherosclerosis, and limiting the blood collection and clinical interview window to the first 4 days of hospitalization. The results of the regression analysis are presented in Table 4. Only NLRP3 had a significant contribution to the likelihood of depression analyzed with age, body mass index, diabetes comorbidity, use of additional medication, Gensini score, duration between myocardial infarction, and blood collection as covariates (*p* = 0.015, OR = 1.72, and CI = 1.11-2.66). However, no statistically significant results were found in the analyses for IL-1β, IL-18, and *hs*CRP.

## 4. Discussion

The main aim of this study was to investigate the possible role of the NLRP3 inflammasome and *hs*CRP, which are thought to play a role in depression and AMI comorbidity. In this study, NLRP3 levels were significantly lower in the healthy control group than in the MI and MID groups, but there was no significant difference between the MI and MID groups. However, the likelihood of depression increased with increasing NLRP3 levels in myocardial infarction patients. 

The NLRP3 inflammasome complex, which is considered a possible common mechanism in the presence of systemic diseases and comorbid depression, has not been previously evaluated in a clinical sample examining the association of AMI and depression. NLRP3 is an intracellular multiprotein complex responsible for innate immune processes. NLRP3 activation has been hypothesized to bridge CVD and depression. Preclinical and clinical studies proved the role of the NLRP3 inflammasome in MDD [24,31,36,37]. Also, clinical studies within the cardiovascular field elucidated the role of the NLRP3 inflammasome following ischemic, nonischemic, acute, and chronic insults to the cardiovascular system [33]. Treatment with several molecules such as oridonin, MCC950, and CT-09 preserved post-MI cardiac functions and limited the infarct size in mice by inhibiting the NLRP3 inflammasome [47].

In our study, the mean score of BDI in the MID group was 18.08 ± 4.57, which indicates mild-to-moderate depression. Although the risk of CVD and mortality increases in the presence of depression and even in the presence of subthreshold depressive symptoms [48,49], based on the results of our study, it should be emphasized that the patients were not primarily included in this study due to depressive complaints; depressive symptoms were detected during the admission for STEMI. This explains why the depression scale scores of our patients were considerably lower than the BDI scores (41.5 ± 8.3) in the study of Alcocer-Gomez et al., which demonstrated increased levels of the NLRP3 inflammasome in depressive patients [36]. A recent study by Taene et al. found that depressed patients had significantly higher NLRP3 mRNA expressions compared to the untreated group, but the decrease in the treated group was not statistically significant [37]. In that study, the mean BDI score of patients was 36.42 ± 10.90 in the untreated group and 24.47 ± 15.64 in the treated group. Even the treated group in their study had higher depression scores than our depression group, and the inadequate treatment response might be one of the reasons for the insignificant result. In our study, the absence of significant difference (*p* > 0.05) in inflammatory marker levels between the MI and MID groups was possibly related to the presence of mild-to-moderate depression. We propose that NLRP3 may be an important marker in the association between depression and MI, but this difference may become statistically significant in a larger sample and with more severe depression. The pathological mechanism of mild-to-moderate depression might be different from that of severe depression. A recent study found that the NLRP3 level in subjects with “reactive” depression (occurring as the result of a stressor) was significantly lower than that of those subjects diagnosed with “endogenous” depression (occurring in the absence of stress) and in healthy controls [50]. In our study, to eliminate the psychological stressors related to myocardial infarction and hospitalization, the interviews were held in the early days of hospitalization, and patients were diagnosed with depression if they fulfilled the depression criteria within a 2-week period.

In the study by Jalil et al., CRP levels were 31 (3–150) mg/L in the MI group and 2 (2–5) mg/L in the control group [34]. In our study, *hs*CRP levels were 4.13 (0.6–6.29) mg/L in the control group, 18.14 (0.64–83.97) mg/L in the MI group, and 25.29 (2.87–229.5) mg/L in the MID group. Although the levels in the MI and MID groups were significantly higher than in the healthy controls, there was no statistical significance between the MI and MID groups. The AHA recommends measurement of the *hs*CRP level to determine cardiovascular risk, and *hs*CRP is categorized as <1 mg/L indicates low risk, 1–3 mg/L indicates moderate risk, and >3 mg/L indicates high risk [14]; thus, *hs*CRP is considered to be a marker for CVD. In a 6-year follow-up study of 82,544 individuals without known CVD, 714 new MI cases were identified, and it was shown that individuals with higher baseline *hs*CRP values had a significantly increased risk for MI at follow-up (*p* < 0.001) [51]. In the same study, it was calculated that every 1 mg/L increase in *hs*CRP level per year increases the risk of future MI by 9.3%. A population-based study evaluated depressive symptoms and perceived stress with metabolic parameters and *hs*CRP and showed that higher *hs*CRP levels were associated with higher perceived stress levels [52]. In contrast, a subgroup of patients diagnosed with somatic symptom disorders demonstrated an association between lower *hs*CRP levels and reduced levels of depression, anxiety, pain, and somatic symptoms [53].

At the same time, *hs*CRP was measured in many psychiatric diseases, and meta-analyses showed that *hs*CRP levels are higher in depression than in healthy controls, which supports the inflammation hypothesis of depression [54]. However, we found no significant difference between the MI and MID groups in terms of *hs*CRP levels. 

In our healthy control group, the mean age was 47.8 ± 5.5 years, the mean BMI was 27.2 ± 2.9 kg/m^2^, and the *hs*CRP levels were 4.13 (0.6–6.29) mg/L. Although the cut-off NLRP3 level in the aforementioned study was recommended as 0.75 ng/mL, the NLRP3 levels of our control group were 0.83 (0.34–1.22) ng/mL. Thus, we might assume that at least some subjects in our healthy control group may have had cardiovascular risk factors. 

Like CVD, diabetes is a chronic disease in which inflammation plays a role in its pathophysiology, comorbidity with depression is common, and NLRP3 is thought to play a role [16,55]. A study conducted by Li et al. demonstrated that patients with diabetic STEMI exhibited higher expression levels of pyruvate kinase M2 (PKM2), NLRP3, IL-1β, and IL-18 compared to patients with non-diabetic STEMI and stable coronary heart disease [56]. The authors hypothesized that the activation of the PKM2-mediated NLRP3 inflammasome through hyperglycemia could potentially enhance plaque vulnerability. However, MI is a macrovascular complication of diabetes, and diabetes and MI co-occur at a high rate, so the presence of concomitant diabetes was not exclusionary in the present study. When the inflammatory parameters were re-evaluated according to the presence of concomitant diabetes, lower levels of IL-18 and *hs*CRP were detected in the presence of diabetes, but no significant change was observed in the NLRP3 and IL-1β levels. This is inconsistent with the findings of the study conducted by Li et al., which suggested that inflammatory parameters are higher if MI and diabetes co-occur compared to patients with MI only. In our study, when patients with a diagnosis of diabetes were evaluated, it was noted that the majority of them were using metformin, as monotherapy or in combination with sitagliptin, or insulin, and we assume that the lower levels of IL-18 and *hs*CRP might be related to this treatment. Metformin is frequently used in the treatment of insulin resistance and type 2 diabetes. In a randomized controlled study, metformin caused a significant decrease in CRP and IL-6 levels compared to baseline levels after a 16-week treatment period [57]. In a meta-analysis, serum CRP and *hs*CRP levels were found to significantly decrease after metformin treatment [58]. This finding is compatible with the data obtained in our study. The number of clinical studies addressing the role of the NLRP3 inflammasome in diabetes is very limited, but preclinical studies show a possible role of the NLRP3 pathway in the pathophysiology of type 2 diabetes [55,59]. Antidiabetic agents might exert an effect on NLRP3 [60,61,62]. However, it is noteworthy that in our study lower levels of IL-18 and *hs*CRP were observed in diabetic patients, but the same effect was not observed for theNLRP3 and IL-1β levels. 

We were not able to detect a concurrent contribution of IL-1β and IL-18 to the likelihood of depression, possibly because of their involvement in the NLRP3-independent pathways [63]. Analyzing gene expressions in conjunction with the serum levels of the inflammatory parameters, while also assessing the other components of the pathway and inflammasome complexes, such as toll-like receptors, ASC, pro-caspase-1, caspase-1, pro-IL-1β, and pro-IL-18, would have provided a better understanding of the pathway and its role in depression–myocardial infarction comorbidity.

Our study had several limitations. First, the patients were not admitted primarily due to depressive complaints; depressive complaints were detected during the admission for STEMI. Second, the study subjects were limited to patients with mild-to-moderate depression. Third, our study was cross-sectional, and it was not possible to evaluate the relationship between CVD and depression from the perspective of causality. Fourth, we did not re-evaluate the patients at another time point to investigate whether the presence of depression or the higher baseline NLRP3 levels predicted worse clinical outcomes. Fifth, since NLRP3 is a sensor capable of detecting a broad range of signals and is associated with many molecular pathways, it was not possible to exclude all potential confounding parameters. Sixth, we aimed to reduce heterogeneity by solely focusing on STEMI patients within the patient group. Nevertheless, a more comprehensive perspective of the data could be achieved by including stable coronary heart disease and non-STEMI patients as well.

The NLRP3 inflammasome remains a novel biomarker in clinical practice, still lacking well-established cut-off values for various clinical conditions. However, it is intriguing to hypothesize that NLRP3 could potentially serve as a candidate for risk assessment for both depression and AMI. Furthermore, the inhibition of NLRP3 inflammasome may have the potential to reduce the risk of depression after AMI, particularly within the subgroup exhibiting elevated baseline levels of NLRP3, leading to improvements in cardiovascular outcomes. It is imperative to test this hypothesis when designing prospective studies.

## 5. Conclusions

This study is the first clinical study investigating the possible role of the NLRP3 inflammasome in comorbid AMI with depression. The increase in NLRP3 levels and the increased likelihood of depression in patients with myocardial infarction can be considered as an important starting point for a possible common nexus between AMI and depression. It is important to highlight that longitudinal studies are necessary to comprehensively assess the bidirectional relationship between AMI and depression.

## Figures and Tables

**Table 1:** Clinical characteristics of participants.

	Control ^a^ (*n* = 20)	MI ^b^ (*n* = 44)	MID ^c^ (*n* = 24)	*p*	Post Hoc
Age (year)	47.2 ± 5	48.3 ± 5.1	47.3 ± 6.5	0.658	
Sex					
Female	5 (25)	3 (6.8)	2 (8.3)	0.142	
Male	15 (75)	41 (93.2)	22 (91.7)		
BMI (kg/m^2^)	27.2 ± 2.9	28.7 ± 3.8	26.8 ± 4.2	0.085	
Type 2 diabetes	0 (0)	8 (18.2)	9 (37.5)	**0.004**	**b > a** **c > a**
Hypertension	0 (0)	3 (6.8)	3 (12.5)	0.402	
Additional medication	0 (0)	6 (13.6)	7 (29.2)	**0.023**	**b > a** **c > a**
Depression history	0 (0)	2 (4.5)	8 (33.3)	**0.001**	**c > b** **c > a**
Smoking					
Non-smoker	9 (45)	3 (6.8)	3 (12.5)	**0.002**	**a > b** **a > c**
Ex-smoker	0 (0)	2 (4.5)	3 (12.5)		
Smoker	11 (55)	39 (88.6)	18 (75)		
HDRS	Null	1.72 ± 1.93	13.75 ± 2.98	**<0.001**	
BDI	Null	3.13 ± 2.75	18.08 ± 4.57	**<0.001**	
SDS	Null	1.27 ± 2.59	9.20 ± 5.00	**<0.001**	
PSS	Null	19.3 ± 7.89	37.04 ± 6.52	**<0.001**	

Note: Findings were shown as mean ± SD or *n* (%). ANOVA, Kruskal–Wallis test, Fisher’s exact test. Different small exponential letters (a, b, c) in a row indicate a statistically significant difference among groups in post hoc tests. HDRS: Hamilton Depression Rating Scale; BDI: Beck’s Depression Inventory; SDS: Sheehan Disability Scale; PSS: Perceived Stress Scale; BMI: body mass index; MI: myocardial infarction; MID: myocardial infarction with depression.

**Table 2:** Laboratory and angiographic characteristics of groups.

	Control ^a^ (*n* = 20)	MI ^b^ (*n* = 44)	MID ^c^ (*n* = 24)	*p*	Post Hoc
WBC (×10^3^/µL)	6.8 ± 1.8	10.2 ± 2.1	12.0 ± 1.8	**<0.001**	**c > b** **c > a** **b > a**
HGB (g/dL)	13.47 ± 1.29	15.23 ± 1.78	14.43 ± 1.4	**0.026**	**b > a**
PLT (×10^3^/µL)	255 (171–454)	215 (152–448)	273.5 (159–522)	0.063	
BUN (mg/dL)	11.5 (6–22)	15 (9–27)	14.5 (9–35)	**0.018**	**b > a**
Creatinin (mg/dL)	0.62 (0.4–1)	0.88 (0.53–1.24)	0.83 (0.6–1.81)	**0.001**	**b > a** **c > a**
AST (U/L)	21 (13–51)	31.5 (18–173)	36 (18–162)	**0.001**	**b > a** **c > a**
ALT (U/L)	24 (9–96)	25 (11–101)	25.5 (13–66)	0.879	
Total cholesterol (mg/dL)	181 (134–285)	191.5 (137–302)	165 (112–316)	0.170	
Triglyceride (mg/dL)	95 (48–144)	139 (46–722)	132 (49–414)	**0.002**	**b > a** **c > a**
HDL (mg/dL)	50.45 ± 13.03	39.68 ± 9.18	39.23 ± 7.06	**0.001**	**a > b** **a > c**
LDL (mg/dL)	113.6 ± 25.24	127.39 ± 36.64	113.82 ± 41.42	0.270	
CK-MB at admission (µg/L)	Null	8.87 ± 12.45	23.66 ± 65.70	0.602	
Troponin at admission (ng/L)	Null	103.55 ± 191.30	344.30 ± 810.09	0.915	
Peak CK-MB (µg/L)	Null	167.76 ± 105.21	156.66 ± 108.26	0.607	
Peak troponin (ng/L)	Null	3370.95 ± 2962.98	3810.60 ± 3225.22	0.743	
NT-ProBNP (ng/L)	Null	994.45 ± 2006.31	1507.95 ± 1917.52	0.123	
Gensini score	Null	59.20 ± 28.8	59.43 ± 25.0	0.778	
Duration between MI and blood collection (day)	Null	1.77 ± 0.88	2.16 ± 1.00	0.105	

Note: Findings were shown with mean ± SD or median (min–max). ANOVA, Kruskal–Wallis test. Different small exponential letters (a, b, c) in a row indicate a statistically significant difference among groups in post hoc tests. WBC: white blood cells; HGB: hemoglobin; PLT: platelet; BUN: blood urea nitrogen; AST: aspartate aminotransferase; ALT: alanine aminotransferase; HDL: high-density lipoprotein; LDL: low-density lipoprotein; NT-ProBNP: N-terminal-prohormone B-type natriuretic peptide; MI: myocardial infarction; MID: myocardial infarction with depression.

**Table 3:** Comparison of inflammatory parameters of the groups.

	Control ^a^ (*n* = 20)	MI ^b^ (*n* = 44)	MID ^c^ (*n* = 24)	*p*	Post Hoc
NLRP3 (ng/mL)	0.83 (0.34–1.22)	0.95 (0.06–4.68)	1.54 (0.34–5.6)	**0.02**	**c > a** **b > a**
IL-1β (pg/mL)	6.17 (3.39–7.73)	13.98 (4.89–20.3)	14.47 (3.31–20.49)	**<0.001**	**c > a** **b > a**
IL18 (pg/mL)	156.23 (128.9–253.93)	289.25 (124.51–586.48)	260.76 (142.62–669.79)	**<0.001**	**c > a** **b > a**
*hs*CRP (mg/L)	4.13 (0.6–6.29)	18.14 (0.64–83.97)	25.29 (2.87–229.5)	**<0.001**	**c > a** **b > a**

Note: Findings were shown with median (min–max). Kruskal–Wallis test. Different small exponential letters (a, b, c) were used in each row to indicate from which group the statistically significant difference among groups occurred in post hoc tests. NLRP3: NOD-like receptor protein 3; *hs*CRP: high-sensitivity C-reactive protein; MI: myocardial infarction; MID: myocardial infarction with depression.

**Table 4:** The relationship among NLRP3, IL-1β, IL-18, and hsCRP levels and the likelihood of depression before and after adjusting for covariates.

	Model 1 ^a^		Model 2 ^b^	
	*p*	OR (95% CI)	*p*	OR (95% CI)
NLRP3	**0.037**	1.46 (1.03–2.09)	**0.015**	1.72 (1.11–2.66)
IL-1β	0.393	1.05 (0.93–1.20)	0.368	1.06 (0.92–1.23)
IL-18	0.649	0.99 (0.99–1.00)	0.952	1.0 (0.99–1.01)
*hs*CRP	0.182	1.01 (0.99–1.03)	0.080	1.025 (0.99–1.05)

Note: binary logistic regression analysis with only STEMI patients. ^a^ Model 1 is unadjusted; ^b^ Model 2 is adjusted for age, body mass index, diabetes comorbidity, use of additional medication, Gensini score, duration between myocardial infarction, and blood collection. OR: odds ratio; CI: confidence interval; NLRP3: NOD-like receptor protein 3; *hs*CRP: high-sensitivity C-reactive protein.

## Data Availability

The data presented in this study are available on request from the corresponding author.

## Supplementary Materials

The following supporting information can be downloaded at https://www.mdpi.com/article/10.3390/jpm13091295/s1, Table S1: Medication history for diabetes patients; Table S2: The correlation between inflammatory parameters and the scores of depression, anxiety, perceived stress, disability, Gensini score in patients with MI and MID; Table S3: The correlation between inflammatory parameters and the scores of depression, anxiety, perceived stress, disability, Gensini score in patients with MI and MID (Type 2 Diabetes was excluded for the analysis); Table S4: The comparison of inflammatory parameters in the presence of Type 2 diabetes.
