# Supplementary material for: The Possible Role of NLRP3 Inflammasome in Depression and Myocardial Infarction Comorbidity
Source: J Pers Med. 2023 Aug 25;13(9):1295. doi: 10.3390/jpm13091295 (PMC10533058; doi:10.3390/jpm13091295)
Supplement: Supplementary file 1 [file jpm-13-01295-s001.zip › jpm-2536360-supplementary.pdf]

Supplementary Table S1. Medication history for diabetes patients

MI (n=8)		MID (n=9)	
**Medication**	**n**	**Medication**	**n**
Insulin	2	Insulin	3
Metformin	1	Metformin	3
Metformin+Sitagliptin	3	Metformin+Sitagliptin	1
None	2	None	2

MI: myocardial infarction; MID: myocardial infarction with depression

Supplementary Table S2. The correlation between inflammatory parameters and the scores of depression, anxiety, perceived stress, disability, Gensini score in patients with MI and MID

	MI								MID							
	**NLRP3**		**IL-1β**		**IL-18**		***hs*CRP**		**NLRP3**		**IL-1β**		**IL-18**		***hs*CRP**	
**Variables**	**r**	**p**	**r**	**p**	**r**	**p**	**r**	**p**	**r**	**p**	**r**	**p**	**r**	**p**	**r**	**p**
**HDRS**	-0,241	0,115	-0,072	0,641	-0,019	0,902	-0,041	0,792	0,251	0,237	-0,048	0,822	0,120	0,577	-0,225	0,291
**BDI**	-0,061	0,696	-0,033	0,834	-0,068	0,663	-0,052	0,735	0,200	0,348	-0,163	0,447	0,162	0,450	-0,133	0,534
**Sheehan**	0,013	0,933	-0,151	0,328	0,131	0,397	-0,079	0,608	-0,340	0,104	-0,222	0,298	-0,023	0,914	-0,132	0,537
**PSS**	0,229	0,135	0,267	0,080	-0,031	0,842	0,125	0,417	-0,123	0,568	-0,204	0,340	0,297	0,159	0,028	0,897
**Gensini**	-0,188	0,222	0,014	0,926	-0,139	0,367	-0,292	0,055	-0,066	0,759	0,080	0,711	0,074	0,732	-0,261	0,218

Spearman correlation test. HDRS: Hamilton Depression Rating Scale; BDI: Beck Depression Inventory; SDS: Sheehan Disability Scale; PSS: Perceived Stress Scale

Supplementary Table S3. The correlation between inflammatory parameters and the scores of depression, anxiety, perceived stress, disability, Gensini score in patients with MI and MID **(Type 2 Diabetes was excluded for the analysis)**

	MI								MID							
	**NLRP3**		**IL-1β**		**IL-18**		***hs*CRP**		**NLRP3**		**IL-1β**		**IL-18**		***hs*CRP**	
**Variables**	**r**	**p**	**r**	**p**	**r**	**p**	**r**	**p**	**r**	**p**	**r**	**p**	**r**	**p**	**r**	**p**
**HDRS**	-0,192	0,261	-0,011	0,951	0,013	0,942	0,019	0,913	0,225	0,421	0,276	0,319	0,163	0,561	-0,174	0,535
**BDI**	-0,034	0,843	-0,003	0,985	-0,023	0,892	0,013	0,939	0,253	0,364	0,070	0,804	0,138	0,624	-0,246	0,378
**Sheehan**	0,031	0,859	-0,214	0,209	0,070	0,685	-0,131	0,445	-0,247	0,375	-0,203	0,468	-0,153	0,586	-0,162	0,564
**PSS**	0,218	0,203	0,251	0,140	-0,036	0,834	0,090	0,603	-0,149	0,597	-0,499	0,058	0,197	0,481	0,032	0,909
**Gensini**	-0,198	0,247	0,048	0,780	-0,085	0,623	-0,268	0,114	-0,129	0,648	0,142	0,612	0,073	0,795	-0,021	0,940

Spearman correlation test. HDRS: Hamilton Depression Rating Scale; BDI: Beck Depression Inventory; SDS: Sheehan Disability Scale; PSS: Perceived Stress Scale

Supplementary Table S4. The comparison of inflammatory parameters in the presence of Type 2 diabetes

	**MI**			**MID**		
	**No (n:36)**	**Yes (n:8)**	**p**	**No (n:15)**	**Yes (n:9)**	**p**
**NLRP3**	0.96 (0.11-4.68)	0.91 (0.06-3)	0.643	2.5 (0.46-5.6)	1 (0.34-4.11)	0.174
**IL-1β**	13.98 (4.89-20.3)	13.98 (5.29-16.74)	0.637	14.57 (3.31-20.49)	13.78 (8.65-18.13)	0.446
**IL-18**	298.37 (131.3-586.48)	267.59 (124.51-352.05)	0.234	299.51 (142.62-669.79)	185.72 (149.42-326.91)	**0.035**
***hs*-CRP**	21.24 (0.64-83.97)	12.62 (6.34-18.89)	0.092	35.45 (10.72-229.5)	11.05 (2.87-49.49)	**0.021**

Note: Findings were shown with median (min-max). NLRP3: NOD-like receptor protein 3; hsCRP: high sensitivity C-reactive protein; MI: myocardial infarction; MID: myocardial infarction with depression
